# Incidence and predictors of mortality among low birth weight neonates in the first week of life admitted to the neonatal intensive care unit in Northwestern Ethiopia comprehensive specialized hospitals, 2022. Multi-center institution-based retrospective follow-up study

**DOI:** 10.1186/s12887-023-04319-0

**Published:** 2023-09-27

**Authors:** Wubet Tazeb Wondie, Kassaye Ahmed Zeleke, Chalachew Adugna Wubneh

**Affiliations:** 1 Department of Pediatrics and Child Health Nursing, College of Medicine, and Health Science, Ambo University, Ambo, Ethiopia; 2 Department of Neonatal Health Nursing, School of Nursing, College of Medicine, and Health Science, University of Gondar, Gondar, Ethiopia; 3 Department of Pediatrics and Child Health Nursing, School of Nursing, College of Medicine, and Health Science, University of Gondar, Gondar, Ethiopia

**Keywords:** First week of life, Incidence, Mortality, Low birth weight, Predictors

## Abstract

**Background:**

Globally, a high number of neonatal mortalities occurs in the first week of life, particularly among low birth weight neonates in low-income countries, including Ethiopia. However, there is limited evidence on the early neonatal mortality of low-birth-weight neonates in Ethiopia. Therefore, this study aimed to assess incidence and predictors of mortality among low-birth-weight neonates in their first week of life admitted to the neonatal intensive care unit in Northwestern Ethiopia Comprehensive Specialized Hospitals, 2022.

**Methods:**

A multi-center retrospective follow-up study was conducted from March 21, 2020 to March 1, 2022, among 761 early neonates with low birth weight admitted in Northwestern Ethiopia Comprehensive Specialized Hospitals. The study participants were selected using simple random sampling technique. Data were collected using a data abstraction checklist ,and checked for completeness and entered into EPI data version 4.6, then exported to STATA 14 for analysis. Kaplan Meier failure curve and log-rank test were used to estimate and compare the probability of death. Both bivariable and multivariable Weibull regression models were fitted to identify predictors of mortality. Finally, a hazard ratio with 95% CI was computed, and variables having a p-value < 0.05 were considered as a significant predictor of mortality.

**Results:**

The incidence of mortality among low birth weight neonates in their first week of life was 75.63 per 1000 neonate day observation (95% CI: 66.76–85.67), preeclampsia (AHR = 1.77;95% CI:1.32-2.36s), perinatal asphyxia (AHR = 1.64; 95% CI:1.14–2.36), respiratory distress syndrome (AHR = 1.76 95% CI;1.31–2.34), necrotizing enterocolitis (AHR = 2.78 95% CI;1.79–4.32), prematurity (AHR = 1.86; 95% CI:1.30–2.67), and birth weight < 1000gram (AHR = 3.13;95% CI: 1.91–5.12) and 1000–1499 gram (AHR = 1.99; 95% CI:1.47–2.68) were predictors.

**Conclusion:**

The incidence of early neonatal mortality in low birth weight neonates was incredibly higher than the overall early neonatal mortality in Northwest Ethiopia (Amhara region). Preeclampsia, perinatal asphyxia, respiratory distress syndrome, necrotizing enterocolitis, prematurity, and birth weight were predictors of mortality. Therefore, stakeholders shall give early identification and emphasis on preventable and treatable predictors. Furthermore, the health care provider shall give education about the importance of breastfeeding, and Antenatal and postnatal care.

**Supplementary Information:**

The online version contains supplementary material available at 10.1186/s12887-023-04319-0.

## Background

Globally, three-fourths of neonatal mortality occurs in the first week of life [1]. A high number of Early Neonatal Mortality (ENM) occurs among Low Birth weight (LBW) neonates [2]. These neonates are 20 times more likely to die than normal birth-weight neonates [3, 4]. Early Mortality among LBW neonates is continued to be a major public health problem, particularly in middle-income, and low-income countries [5]. According to World Health Organization (WHO) report, in 2020, above 2.4 million neonates died annually, and 80% of them are LBW [5, 6]. The first week of life is a critical period for the survival of LBW neonates. Of the total neonatal deaths, 75% occurs during the first weeks of life, with one-third of deaths occurring on the first day of birth [1, 6]. Specifically in Southeast Asia, the mortality of LBW neonates ranges from 19 to 31% [7], and in Sub-Saharan Africa, the mortality of these neonates ranges from 21% in females to 23.9% in males [8].

The incidence of ENM among LBW varies across the country with the highest incidence of ENM occurring in low-income countries, for instance in Bangladesh it was 112 per 1,000 neonate day observations, in Brazil 52 per 1,000 neonates [9], in India 21.22 per 1000 live births [10], Burkina Faso 1.93 per 1000 neonate/day [11], and in Bahir Dar, Ethiopia 22.16 per 1000 neonate day observation [12].

Ethiopia aimed to reduce the Neonatal Mortality Rate (NMR) from 29 to 1000 live births in 2016 [13] to 11 per 1000 live births in 2019/2020, including LBW neonates [14]. Despite the government’s aim of reducing Neonatal Mortality (NM), paradoxically it had been increased to 33/1000 live births in 2019, from this, the mortality of LBW neonates is a major public health problem [15]. In Ethiopia, the mortality of LBW neonates ranges from 11.0 to 37.8% [12, 16–18]. Overall, early neonatal mortality in the Amhara region is 43 per 1000 live births, which is the highest figure in the country [13].

These studies suggested that the predictors of mortality among LBW neonates are maternal age [12], Antenatal care(ANC) follow-up [16], neonatal age, residence, sex, type of pregnancy [19, 20], birth weight [9, 19, 21], sepsis [12, 18, 22], perinatal asphyxia [21, 23, 24], Respiratory Distress Syndrome(RDS) [12, 18, 25, 26], prematurity [12, 16, 25, 27–29], Necrotizing Enter Colitis(NEC) [12, 26, 30, 31], 1st, and 5th minute (Appearance, Pulse, Grimace, Activity, Respiration) (APGAR) score [19, 32], place of delivery, preeclampsia [33, 34], maternal diabetes mellitus (DM) [17, 25], and maternal Human Immunodeficiency Virus(HIV) infection [13, 35]. Nevertheless, the incidence and predictors of mortality among LBW neonate, specifically in their first week of life is not fully addressed.

To tackle this problem, and increase the survival of LBW neonates, efforts have been made globally as well as nationally. For instance, WHO is working with the Ministry of Health (MoH) and other partners to expand the quality of services for LBW babies [36]. Interventions like the provision of adequate nutrition for adolescent girls were done to reduce mortality among LBW neonates [37]. In addition, the Sustainable Development Goal (SDG 3) focuses on reducing neonatal mortality to below 12 per 1000 live births by 2030 [38]. Furthermore, Ethiopia accepted initiatives to decrease the mortality of LBW neonates [39], and currently, planned to reduce neonatal mortality from 33 to 1000 live births to 21 per live births by the year 2024/2025 [40].

Despite these efforts and strategies, neonatal mortality remains high [35], and increased from 29 to 33 per 1000 live birth since 2016 [13, 35], Despite this problem, the previous studies conducted in Ethiopia have not focused on the critical period of life before 7 days among LBW neonates, and, there is limited evidence on this subgroup of neonates in the critical period of life regarding the incidence of ENM and its predictors among LBW neonates in the study setting. Therefore, estimating the incidence, and identifying predictors among high-risk groups in the critical period of life are the cornerstones to improve LBW neonate survival, progress toward the national plan, designing appropriate interventions, and providing valuable clinical information for health professionals. Hence, this study aimed to assess the incidence and predictors of mortality among LBW neonates in the first week of life admitted to Comprehensive Specialized Hospitals of Northwest Ethiopiain.

## Methods

### Study setting, design, and period

An institution-based multi-center retrospective follow-up study was conducted using data of LBW neonates from March 21, 2020 to March 1, 2022 (the data were extracted from May 1 to June 26, 2022) in Neonatal Intensive Care Unit (NICU) of Northwest Ethiopia (West Amhara) Comprehensive specialized hospitals. Comprehensive Specialized Hospitals in the Northwest part of Ethiopia (West Amhara region) are the University of Gondar (UoG), Felege hiwot, Tibebe Ghion, Debre Tabor, and Debre Markos. UoGCSH is found in Gondar town, and this hospital has an average annual admission of 726 LBW neonates and 528 of them were admitted in the first week. Felege hiwot and Tibebe Ghion CSH are found in Bahir Dar city. Felege Hiwot CSH has average annual admission of 720 LBW neonates and 504 of them were admitted in the first week. Tibebe Ghion has an average annual admission of 690 LBW neonates, and 484 of them were admitted within the first week. Debre Tabor CSH is found in Debre Tabor town. This hospital has an average annual neonatal admission of 512 LBW neonates, and, 408 of them were admitted in the first week. Debre Markos CSH is found in Debre Markos town, and this hospital has an average annual admission of 612 LBW neonates and 444 of them were LBW early neonates. These Hospitals have NICU with mixed health professionals (neonatal and comprehensive nurses, general practitioners, pediatricians, and other staffs). The major services in the NICU include general neonatal care services, blood and exchange transfusion, phototherapy, and ventilation support such as Continuous Positive Air Pressure (CPAP).

### Study population

All LBW neonates in the first week of life admitted to the NICU of Northwest Ethiopia Comprehensive Specialized Hospitals were considered as the source population, and all low birth weight neonates in the first week of life admitted to the NICU of Northwest Ethiopia Comprehensive Specialized Hospitals from March 21, 2020 to March 1, 2022 were taken as a study population.

### Eligibility criteria

LBW neonates in their first week of life who were admitted to NICU from March 21, 2020, to March 1, 2022, were included, Whereas, Neonates with an incomplete chart (outcome status and charts with one of the following predictor is missed (gestational age, birth weight, neonatal age, sex, type of pregnancy, place of delivery, date of admission, and discharge)) were excluded.

## Sample size estimation and sampling technique

The sample size was determined using STATA 14, using the Cox proportional hazard model by considering the following assumptions: confidence level (95%), Power (80%), a 28.35% Probability of an event, and 1.483 hazard ratio of the predictor (maternal age above 35) [12], 5% Margin of error, and 10% probability of withdrawal (chart attrition). Accordingly, the final estimated sample size was 793 LBW neonates chart. Within two years, the hospitals had a total of 4752 LBW early neonate admissions. Accordingly, the total sample size was proportionally allocated to each hospital, and the neonate’s chart was selected by a simple random sampling technique using a computer-generated random number in Excel.

### Variables of the study

The dependent variable of the study was mortality in the first week of life, whereas the independent variables include (1) socio-demographic variables such as (maternal age, sex of the neonate, age of the neonate, place of delivery, residence). (2) Maternal medical factors such as maternal DM, HTN, HIV/AIDS, TB, Anemia, and other illnesses (STI, UTI). (3) Maternal obstetric factors such as (type of pregnancy, gravidity, parity, PROM, APH, mode of delivery, preeclampsia, ANC follow-up, corticosteroid administration), and (4) Neonate’s clinical related variables such as (PNA, RDS, sepsis, jaundice, hypothermia, hypoglycemia, NEC, 1st & 5th minute APGAR score, breastfeeding, congenital anomalies, IUGR, birth weight, gestational age).

## Operational definition

### Low birth weight

Weight at birth less than 2,500 g regardless of gestational age [3].

### Early neonatal mortality

The death of the newborn from the time of birth up to seven completed days of life [2, 41].

### Event

Death of LBW neonates at a specific time (day) within seven days of age.

### Censored

LBW neonates who left the follow-up (survived over the follow-up period, lost to the follow-up, transferred/ referred to other health facilities, and left against medical treatment).

### Extremely low birth weight

Neonates born with less than 1000 gm of birth weight [6].

### Follow-up time

The time from admission to NICU to either death or censorship occurs.

### Prematurity

Neonate born before 37 completed weeks of gestational age [42].

### Survival status

Outcome of neonates, either death or censored.

### Very low birth weight

Neonates born with (1000–1499 gm) of birth weight [42].

### Data abstraction checklist and procedures

The data abstraction checklist was adapted by reviewing different relevant literature, and guidelines [8, 12, 17, 19, 21, 42–44]. It contains four parts: socio-demographic characteristics of mothers and neonates, maternal medical disorders, obstetric, and gynecological factors, neonate’s clinical-related variables of LBW neonates, and outcome measures. The data were retrospectively collected by 5 experienced BSc nurses and supervised by 4 experienced BSc nurses. The medical registration number of LBW neonates who were admitted in the early neonatal period from March 21, 2020 to March 1, 2022 was first obtained from the FMOH registration book. Then, the required number of neonates’ medical charts was selected by a simple random sampling technique according to eligibility criteria.

### Data quality control

The data abstraction checklist was evaluated by the expert researcher, and then a pretest was done on 5% of the sample size in a similar setting, and necessary amendments were done. One-day training was given to data collectors and supervisors focusing on the purpose of the study, the data collection tool, data collection methods, and ethical concerns during the data collection period. All the collected data were checked for their completeness and consistency by the data collectors and supervisors daily.

### Data processing and analysis

The data were cleaned, coded, and entered into Epi-Data version 4.6.0.6, and the analysis was done using STATA version 14 statistical software. The outcome was dichotomized into death, coded as “1”, and censored coded as “0”. The variance inflation factor was used to assess multi-collinearity. The incidence rate of mortality was calculated for the entire follow-up by dividing the number of new cases of mortality of LBW early neonates by the total person-days of follow-up. Kaplan Meier failure curve was used to estimate the time to death during the follow-up. The log-rank test was employed to compare statistical differences between groups of independent variables. The Schoenfeld residual global test for proportionality assumption was checked, and its (P-value = 0.4282) was used as a suggestive of satisfying the assumption. The log-likelihood and Akaike Information Criteria (AIC) were applied to select the best-fitted model, and a model with minimum AIC was considered a fitted model. Based on this the Weibull regression model with the (AIC = 1196.251) value was the fitted model. The goodness of fit of the model was also checked using the Cox-Snell residual test, and it was close to the bisector. Variables with P-value < 0.25 in the bi-variable analysis were entered into the multivariable Weibull regression model analysis. Crude and adjusted hazard ratios with 95% CI, were used to determine the strength of the association. In multivariable analysis variables having a p-value < 0.05 were considered as predictors of mortality.

## Results

### Socio-demographic characteristics of mother-neonates pair

A total of 793 LBW neonates’ medical charts were reviewed with a completeness rate of 761(95.96%) charts. Of the total included neonates, about half of the participants 395 (51.91%) were male. Above two-thirds, 525 (68.99%) of neonates were admitted within 24 h of birth. Most 537(70.37%) of the mothers were in the age category of 21–34 years old. The median age of the mothers was 28 (IQR 23, 31). (Table 1).

**Table 1:** Socio-demographic characteristics of the neonates and mothers admitted to NICU of Northwest Ethiopia CSH from March 2020 to March 2022 (N = 761)

Variable	Number	Percent	Status	
			Death	Censored
Age of the neonate in days				
≤1 day 2–3 days 4–7 days	525	68.99	186	339
	143	18.79	45	98
	93	12.22	16	77
Sex				
Male Female	395	51.91	118	277
	366	48.09	129	237
Maternal age				
≤20 21–34 ≥35	105	13.57	39	66
	537	70.37	180	357
	119	15.64	28	91
Residence				
Urban Rural	445	58.48	142	303
	316	41.52	105	211
Place of delivery				
Health institution Out-of-health institution	731	96.06	242	489
	30	3.94	5	25

### Maternal obstetric and gynecological-related characteristics

Of the total enrolled mothers in this study, the Majority 696 (91.46%) had ANC follow-ups in nearby health institutions. Nearly three fourth of neonates were delivered via spontaneous vaginal delivery (SVD), and 567(74.51%) were singleton pregnancies. About one-fourth of mothers 203 (26.68%) had preeclampsia, and 185 (24.31%) mothers were taking corticosteroid treatment(Table 2).

**Table 2:** Maternal obstetric and gynecological related characteristics of low birth weight early neonates in Northwest Ethiopia CSH from March 21, 2020 to March 1, 2022 (N = 761)

Variables	Frequency%	Status	
		Death (%)	Censored (%)
ANC			
Yes No	696(91.46) 65 (8.54)	223(90.28) 24(9.72)	473(92.02) 41(7.98)
Type of pregnancy			
Single	567(74.51)	167(67.61)	400(77.82)
Multiple	194(25.49)	80(32.39)	1114(22.18)
Mode of delivery			
SVD Instrumental CS	565(74.24) 29(3.81) 167(21.94)	182(73.68) 5(2.02) 60(24.29)	383(74.51) 24 (4.67) 107(20.82)
Gravidity			
Primi-gravida Multi-gravida	147(19.32) 614(80.68)	40(16.19) 207(83.81)	107(20.82) 407(79.18)
Parity			
Primi-para Multipara	415 (54.53) 346 (45.47)	127(51.42) 120(48.58)	288(56.03) 226(43.97)
Corticosteroid Treatment Yes No	185(24.31) 576(75.69)	71(28.74) 176(71.26)	114(22.18) 400(77.82)
Preeclampsia			
Yes No	203(26.68) 558(73.32)	105(42.51) 142(57.49)	98(19.07) 416(80.93)
PROM			
Yes No	124 (16.29) 637(83.71 )	46(18.62) 201(81.38)	78((15.18) 446(84.82)
APH			
Yes No	71(9.33) 690(90.67)	29(11.74) 218(88.26)	42(8.17) 472(91.83)

ANC: Antenatal care, PROM: Premature rupture of membrane, APH: Antepartum hemorrhage, SVD = Spontaneous vaginal delivery, CS = Cesarean section

### Maternal medical-related characteristics of study participants

Among the total enrolled mothers in this study, 19(2.5%) had chronic hypertension, 39 (5.12%) had HIV/AIDS, and 17(2.23%) had other comorbidities ((Urinary Tract infection (UTI), syphilis, and Hepatitis B Virus (HBV))(Table 3).

**Table 3:** Maternal medical characteristics of low birth weight early neonates admitted to NICU of Northwest Ethiopia Comprehensive Specialized Hospitals from March 21, 2020 to 1 March, 2021(N = 761)

Variable	Frequency (%)	Status	
		Death %	Censored %
**Chronic hypertension**			
Yes No	19 (2.50) 742(97.50)	8(3.24) 239(96.76)	11(2.14) 503(97.86)
**Maternal DM**			
Yes No	11(1.45) 750(98.55)	5(2.02) 242(97.98)	6(1.17) 508(98.83)
**Maternal HIV**			
Yes No	39(5.12) 722(94.88)	21(53.85) 226(31.30)	18(46.15) 496(68.70)
**TB**			
Yes No	13(1.71) 748(98.29)	6(2.43) 241(97..57)	7(1.36) 507(98.64)
**Anemia**			
Yes No	13(1.71) 748 (98.29)	5(2.02) 242 (97.98)	8(1.56) 506(98.44)
**Other comorbidities**			
Yes No	17(2.23) 744(97.77)	6(2.43) 241(95.57)	11(2.14) 503(97.86)

DM: Diabetes mellitus, TB: Tuberculosis, HIV: Human immune deficiency virus

### Clinical related and other characteristics of LBW early neonates

Above two-thirds 512(67.28%) of low birth weightearly neonates were premature, and the median gestational age was 35 weeks with an interquartile quartile range of 5 weeks. Three-fourths of neonates 570(74.90%) weighed between 1500, and 2499 g with a median weight of 1750 ± 600 IQ. The common medical problem among LBW early neonates was sepsis (65.83%), and RDS was the leading cause of death in 167(62.75%). Other common medical problems include jaundice, PNA, NEC, IUGR, hypoglycemia, and congenital anomalies (Table 4).

**Table 4:** Clinical characteristics of low birth weight early neonates admitted to Northwest Ethiopia CSH from March 21, 2020 to March 1, 2022. (N = 761)

Variable	Frequency %	Status	
		Died %	Censored%
Birth weight in gram			
≤ 999 1000–1499 1500–2499	33(4.34) 158(22.86) 570(74.90)	23(9.31) 97(39.27) 127(51.42)	10(1.95) 61(11.87) 443(86.19)
Gestational Age in week			
<37 ≥37	512(67.28) 249(32.72)	203(82.19) 44(17.81)	309(60.12) 205(39.88)
First-minute APGAR score			
<7 ≥7	272(35.74) 489(64.26)	123(49.80) 124(50.20)	149(28.99) 365(71.0)
Fifth-minute APGAR score			
<7 ≥7	71(9.33) 690(90.67)	47(19.03) 200(80.97)	24(4.67) 490 (95.33)
Breastfeeding			
Yes No	712(93.56) 49(6.44)	228(92.31) 19(7.69)	484 (94.16) 30 (5.84)
PNA			
Yes No	87(11.43) 674(88.57)	47(19.03) 200(82.19)	40(7.78) 474(92.22)
Sepsis			
Yes No	501(65.83) 260(34.17)	162(67.61) 85(32.39)	339(65.95) 175(34.05)
RDS			
Yes No	356(46.78) 405(53.22)	167(62.75) 80(37.25)	189(36.77) 325(63.23)
Jaundice			
Yes No	104(13.67) 657(86.33)	22(8.91) 225(91.09)	82(15.95) 432(84.05)
NEC			
Yes No	46(6.04) 715(93.96)	26(10.53) 221(89.47)	20(3.89) 494(96.11)
IUGR			
Yes No	41(5.39) 720(94.61)	19(7.69) 228(92.31)	22(4.28) 492(95.72)
Congenital anomalies			
Yes No	29(3.81) 732(96.19)	7(2.83) 240(97.17)	22(4.28) 492(95.72)
Hypothermia			
Yes No	269(35.35) 492(64.65)	101(40.89) 146(59.11)	168(32.68) 346(67.32)
Hypoglycemia Yes	29(3.81)	12(4.86)	17(3.31)
No	732(96.19)	235(95.14)	497(96.69)

PNA = perinatal Asphyxia, APGAR = Appearance, Pulse, Grimace, Activity, Respiration, NEC = Necrotizing enterocolitis, RDS = respiratory distress syndrome, IUGR = Intrauterine growth restriction

.

### Incidence of mortality in the first week of life and overall survival outcome of the follow-up among LBW neonates

In this study, a total of 761 LBW early neonates were followed for up to 7 days of age starting from the date of admission. Among those Low birth weight neonates in the first week of life, 247(32.46%) died, 170 (22.34%) were discharged with improvement, 5(0.66%) transferred to other health institutions, 6(0.79%) lost follow-up, 15(1.97%) left against medical advice and 318 (41.79%) were surviving beyond the follow-up period. Most 80 (32.3%) of neonates died within the first day after admission, 47(19.03%) on the second day, 46 (18.2%) on the third day, 27(10.93%) on the fourth day, 27(10.93%) in the fifth day, 13(5.26%) in the sixth day, and 7(2.83%) were died in the seventh day after admission. The total neonate-day observation during the entire follow-up time was 3266 person-days.

In this study, the overall incidence of early neonatal mortality was 75.63 per 1000 neonate day observations (95% CI: 66.76–85.67). The incidence of mortality at the end of the first day was 105.12 (95% CI; 84.44 -130.88), on the second day 73 0.09 (95%CI: 54.91–97.28), on the third day 84.24(95% CI: 63.10-124.47) on the fourth day 61.22 (95% CI: 41.98–89.27), on the fifth day 74.58 (95% CI: 51.14–108.7), on the sixth day 44.52(95% CI:25.85–76.67), and on the seventh day 31.16(95%CI: 15.10-66.44) per 1000 neonate. Similarly, the incidence rate of mortality among ELBW was 223.30 (95% CI; 148.38-336.03), among VLBW, it was 156.70 (95% CI; 128.43–191.20), and among LBW (1500-2499gm) neonates, 49.92 (95% CI; 41.95–59.40) was observed per 1000 neonate days’ observation period.

## Overall failure function

**Fig. 1:**
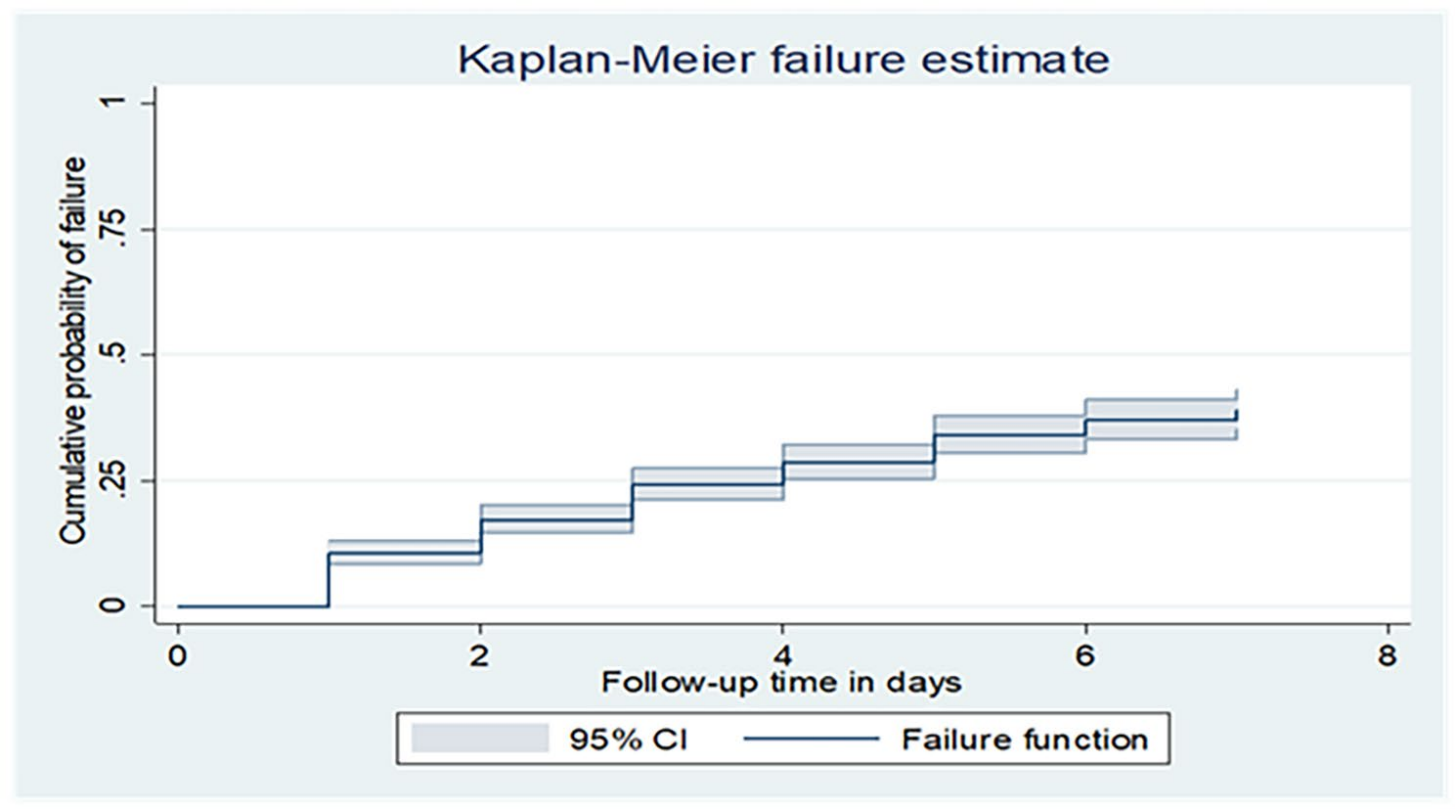
The overall Kaplan Meier failure estimate of mortality among low birth weight in the first week of life in Northwest Ethiopia Comprehensive Specialized Hospitals, from March 21, 2020, to March 1, 2022

*The mean time to mortality of the entire cohort was 5.48 (95% CI: 5.32–5.65) days. The cumulative probability of death at the end of the 1st day was 0.108 (0.088–0.133), at the end of the 3rd day, it was 0.25 (0.22–0.28), and at the end of the 7th day, it was 0.423(0.38, 0.47).In this study, as hospital stay increases, the hazard of death increases, and the survival probability was decreased (*Fig. 1*).*

### Comparison of failure function for different categorical variables

**Fig. 2:**
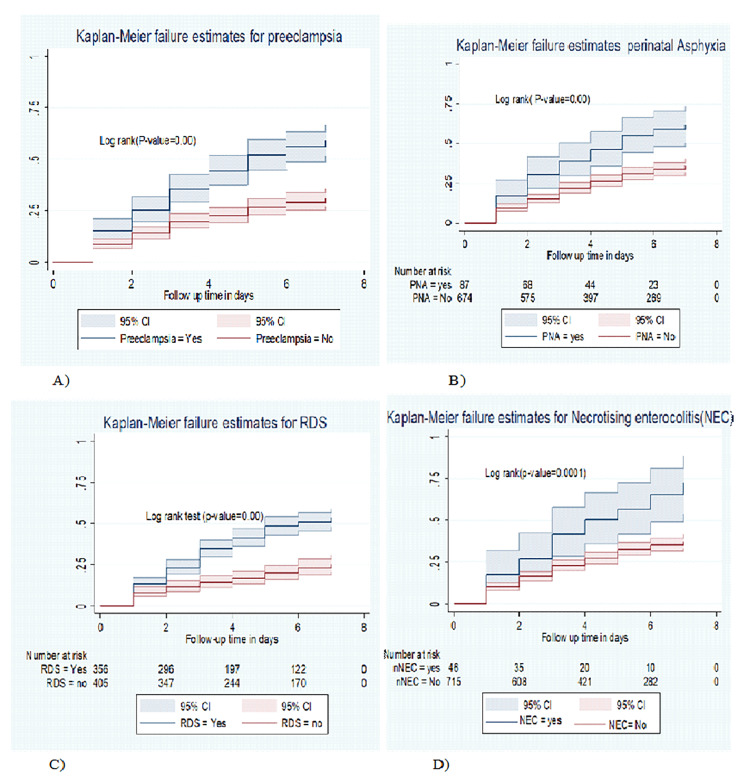
The Kaplan Meier failure estimates of low birth weight early neonates, by (A) Preeclampsia, (B): PNA, (C): RDS, (D); NEC in Northwest Ethiopia CSH, from March 21, 2020, to March 1, 2022

In this study, neonates born from a mother with preeclampsia had a higher risk of death than neonates born from a mother without preeclampsia. The mean hazard time to death was 4.71 days (Fig. 2A). Similarly, LBW early neonates with PNA had an increased risk of death than LBW neonates who did not have PNA, and the mean time of death was 4.69 days (95% CI 4.17–5.21) as compared with their counterparts (Fig. 2B).

The result of this study also showed that LBW neonates with RDS had lower survival than their counterparts. The mean hazard time to mortality was 5 days (95% CI 4.75–5.25) as compared to neonates without RDS (5.9 days) *(*Fig. 2c). Similarly, LBW neonates with NEC had a higher risk of death as compared with their counterparts. The mean time to death was 4.42 days (95% CI: 3.71–5.13) as compared to LBW neonates without NEC (5.55 days) (Fig. 2D).

In this study, neonates having ELBW (< 1000gm) and VLBW (1000-1499gm) had lower survival time than low birth weight neonates (1500-2499gm). In the present study, nearly all 23 (69.70%) ELBW neonates, and 97 (61.39%) VLBW neonates died. The mean hazard time to death for EVLBW and VLBW was 3.48 days, and 4.38 days respectively, as compared to those LBW neonates with a mean hazard time to death of 5.95 days (Supplementary file 1). In addition, Premature LBW neonates were at high risk of death as compared to those term LBW neonates (Supplementary file 1). These differences were statistically significant with a (P-value ≤ 0.0001) in the log-rank test.

### Proportional hazard assumption test by schoenfeld residuals

The Cox proportional hazard assumption was checked using the Schoenfeld residuals test individually and simultaneously (globally). The test showed that the P-value for each covariate and the whole covariates simultaneously were above 0.05 (P-value = 0.4282).

#### Model comparison and diagnostics

Model comparison of both the semi-parametric and parametric hazard models were done statistically using information criteria (AIC, BIC) to select the most parsimonious model for the data set. Based on this, the Weibull regression model with the **(AIC = 1196.251)** was the parsimonious model (Table 5).

**Table 5:** Model comparisons among the Cox proportional hazard model and parametric Regression models using AIC, BIC, and LR criteria

Model	Baseline hazard	Log-likelihood ratio	AIC	BIC
Cox regression	Unspecified	-1466.71	2977.42	3079.382
Weibull regression	**Weibull**	**-574.1256**	**1196.251**	**1307.482**
Exponential regression	Exponential	-585.0281	1216.056	1322.653
Gompertz regression	Gompertz	-582.7498	1213.5	1324.731

**Fig. 3:**
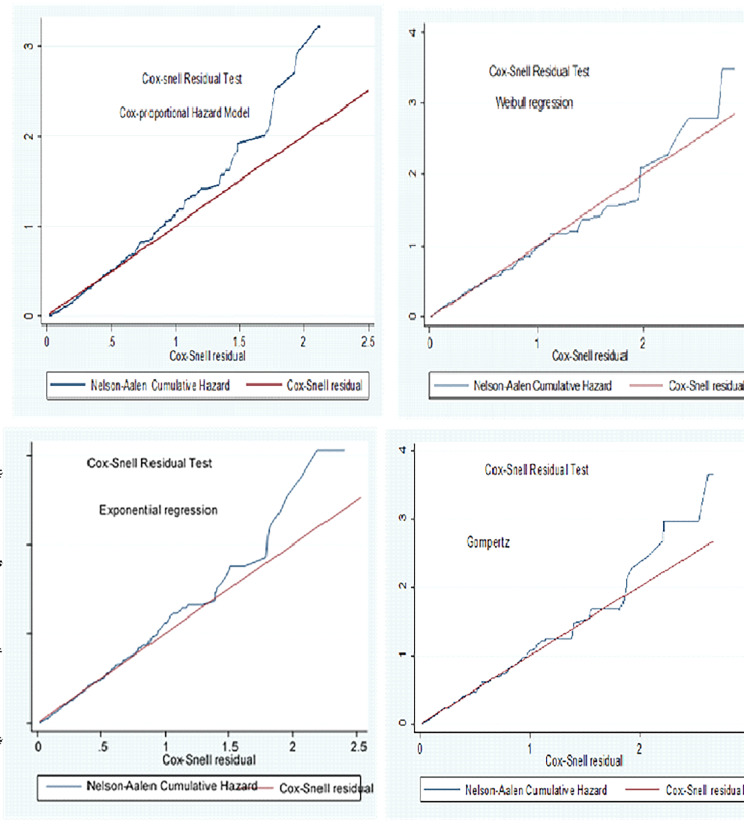
The Cox-Snell residual test of Semi-parametric, and parametric Survival models

The goodness of fit for the fitted model was also checked using the Cox-Snell residual test and as shown in the figure Weibull regression model was adequate (Fig. 3).

### Predictors of mortality

In the bi-variable Weibull regression sex, mode of delivery, preeclampsia, APH, antenatal corticosteroid treatment, gravidity, maternal HIV/AIDS, PNA, RD, jaundice, hypothermia, NEC, IUGR, first, and fifth-minute APGAR score, birth weight, gestational age, maternal age, and type of pregnancy were significantly associated with early mortality (p-value < 0.25). However, in the multi-variable Weibull regression model only Preeclampsia, PNA, RD, NEC, birth weight, and gestational age were significant predictors of early mortality (P-value < 0.05). In this study, LBW neonates born from a mother with preeclampsia had a 1.77 times higher risk of death than their counterparts. (AHR = 1.77; 95%CI: 1.32–2.36). LBW neonates with PNA had a 1.64 times higher risk of death than neonates without PNA (AHR = 1.64, 95%; CI: 1.14–2.36) by keeping other variables constant. Similarly, LBW neonates with RDS had a 1.76 times higher risk of death than LBW neonates without RDS (AHR = 1.76; (95% CI: 1.31–2.34)) holding other variables constant.

The hazard of death in LBW neonates with NEC was 2.78 times higher as compared with their counterparts (AHR = 2.78: 95% CI; 1.79–4.32). LBW neonates with birth weight less than 1000gm. and 1000–1499 gm. were three times and two times at high risk of death with (AHR = 3.13 ;(95% CI: 1.91–5.12)) & (AHR = 1.99; (95%; CI:1.47–2.68)) respectively holding other variables constant. The current study also showed that LBW early neonates who are preterm were 1.86 times (AHR: 1.86 ;( 95% CI; 1.30–2.67)) at higher risk of death as compared to those of term neonates (Table 6).

**Table 6:** Bivariable and multivariable Weibull regression analysis for predictors of mortality among low birth weight neonates in their first week of life admitted to NICU of Northwestern Ethiopia Comprehensive Specialized Hospitals from March 21, 2020 to March 1, 2022. (n = 761)

Variables	Status		CHR(95%CI)	AHR(95%CI)
	Death	Censored		
Sex				
Male Female	118 129	277 237	0.83(0.65-1.065) 1	1.03(0.783–1.35) 1
Maternal age				
≤20 ≥35 21–34	39 28 180	66 91 357	1.2(0.86-1.72) 0.67(0.45 − 1.00) 1	1.41(0.95–2.08) 0.67(0.44–1.02) 1
Mode of delivery				
Instrumental CS SVD	5 60 182	24 107 383	0.41(0.17–0.99) 0.99(0.75–1.34) 1	0 0.70(0.28–1.77) 1.01(0.71–1.44) 1
Type of pregnancy				
Multiple Single	80 167	114 400	1.36(1.04–1.78) 1	1.15(0.86–1.55) 1
Corticosteroid treatment				
No Yes	176 71	400 114	0.83(0.63–1.09) 1	1.07(0.79–1.46) 1
Preeclampsia				
Yes No	105 142	98 416	**2.23(1.73–2.87)** **1**	1.77(1.32–2.36) *** **1**
APH				
Yes No	29 218	42 472	1.40 (0.95–2.06) 1	1.38 (0.92–2.08) 1
Gravidity				
≤1 >1	40 207	117 407	0.79(0.57–1.11) 1	0.82(0.57–1.20) 1
HIV/AIDS				
Yes No	21 226	18 496	1.72(1.10–2.69) 1	1.53(0.95–2.46) 1
1st minute APGAR				
<7	123	149	1.92 (1.49–2.46)	1.32(0.98-1.79)
≥7	124	365	1	1
5th minute APGAR				
<7 ≥7	47 200	24 490	2.56(1.87–3.53) 1	1.40(0.95–2.08) 1
Birth weight				
≤999 1000–1499 1500–2499	23 97 127	10 61 443	**4.72(3.02–7.34)** **3.22(2.47–3.4.20)** **1**	3.13(1.91–5.12) ** 1.99(1.47–2.68)*** **1**
Gestational age				
<37 ≥37	208 39	304 210	**2.6(1.85–3.66)** **1**	1.86(1.30–2.67)** **1**
PNA				
Yes No	47 200	40 474	**2.15(1.56–2.95)** 1	1.64(1.14–2.36) ** 1
RDS				
Yes No	167 80	189 325	**2.6(1.99- 3.40** **1**	1.76(1.31–2.34)*** **1**
Jaundice				
Yes No	22 225	82 432	0.59(0.38-0.92) 1	0.81(0.50–1.29) 1
NEC				
Yes No	26 221	20 494	**2.34(1.56–3.51)** 1	2.78(1.79–4.32)*** 1
Hypothermia				
Yes No	101 146	168 346	1.21(0.94–1.57) 1	1.05(0.80–1.38) 1
IUGR		`		
Yes No	19 228	22 492	1.52 (0.95–2.43) 1	1.58(0.95–2.61) 1

NB: ***=Significant variiable with p-value = 0.00 &**=p-value ≥ 0.001–0.05, 1 = reference

## Discussion

The overall aim of this study was to assess incidence and predictors of mortality among LBW neonates in the first week of life admitted to the NICU of Northwestern Ethiopia Comprehensive Specialized Hospitals. This study showed that at the end of the follow up 32.46% (95% CI 29.12% -35.8%) of LBW neonates died. This finding was in line with a study conducted in South Africa (32%) [22], and Brazil (29%) [19]. The possible reason could be both of the study areas where referral hospitals in which different complicated neonatal cases can be referred from different corners of the country. So, due to a high number of complicated cases, the mortality could be increased. On the other hand, this result was higher than a study conducted in Tanzania (5%) [45], and India 21.2% [21]. This marked difference might be attributed to NICU setup, differences in study design, and inclusion criteria. Those studies from other countries were conducted in a cross-sectional study design. The other reason is those studies include only inborn neonates, but the present study included both inborn and outborn neonates. However, the result of this study was lower than a study conducted in Saudi Arabia (41%) [46]. This difference might be due to the characteristics of the study participants, because they included only extremely low birth weight, and premature neonates [21].

In this study, the overall incidence of early neonatal mortality was 75.63 per 1000 neonate day observations (95% CI: 66.76–85.67). This finding was lower than a study conducted in Bangladesh with 112 per 1000 neonate day observations with (95% CI, 91–136) [47]. This variation might be due to the way of follow-up. Those studies start to follow from the time of birth and in their home up to the end of follow-up, but in the present study, the follow-up starts from admission to NICU and did not include neonates who died immediately after birth, and in their homes, so this may underestimate the overall incidence of ENM. In contrast to these, the finding of this study was higher than a study conducted in Brazil 52 per 1000 neonates [9], India 21.22 per 1000 live births [10], Burkina Faso 1.93 per 1000 neonate /day (95% CI, 1.2–3.1) [11], and Bahir Dar 22.16 (95% CI14.14-34.75) per 1000 neonate day observation at the seventh day [12]. The variation between Brazil, Burkina Faso, and the present study might be due to differences in inclusion criteria, quality of service provision, and NICU setup differences. Those studies included LBW neonates who were not admitted, whereas the present study included only admitted neonates. Additionally, hospitals in Brazil and Burkina Faso may have more advanced care and service provision, and their NICU setup may be more advanced than hospitals of our study setting.

In this study, the incidence of mortality on the first day was high, with an incidence of 105.12 (95% CI; 84.44 -130.88) per 1000 neonate days’ observation. This finding was nearly three times higher than a study conducted in Bahir Dar, Ethiopia 38 per 1000 neonate day observations [12]. The possible reason for this discrepancy might be that, in the present study, the proportion of LBW neonates with RDS and LBW neonates from preeclampsia mothers was higher than a study conducted in Bahir Dar. Additionally, it could be due to differences in neonates’ age that include only neonates who were in critical time (the first week of life). The other possible explanation might be related to pregnancy and birth-related complications that may result in a delay in the diagnosis and management of neonatal problems among early neonates.

Concerning predictors of mortality, this study identified several predictors of mortality. Accordingly, LBW neonates born from a mother with preeclampsia were nearly two times more likely to die than neonates whose mothers did not have preeclampsia. This result was consistent with a study done in Greece [34], and China [33]. This is due to a decrease in uteroplacental blood flow, and then, the neonates develop hypoxia and IUGR which could increase the risk of dying [48]. Additionally, preeclampsia can be a threat to maternal life, and in this case, terminating the pregnancy could be mandatory, and therefore, the neonate would be delivered with immature organ systems, which increases the risk of death.

In addition, LBW neonates with perinatal asphyxia (PNA) had a higher risk of early death as compared with neonates without PNA. This result was consistent with studies done in Brazil [24], India, and Bangladesh [21, 23]. The possible reason for this could be the PNA contribut of the deprivation of oxygen in the body, which leads to progressive hypoxemia, and hypercapnia resulting in the central nervous system and other end-organ damage [42]. In the current study, the hazard of death among neonates with RDS was 1.76 times higher as compared with its counterpart. This finding was in line with a study conducted in Brazil [49], Zimbabwe [25], India [21, 44], and Bahir Dar, Ethiopia [12]. Since the majority of neonates are premature, the problem of lung immaturity (lack of adequate surfactant) is a common phenomenon that leads to lung collapse, and respiratory failure [42]. Furthermore, it might be due to a lack of administration of antenatal corticosteroids to mothers before 37 weeks of gestation, lack of surfactant replacement therapy [50], and due to inadequate number and lack of trained personnel in the use of Nasal Continuous Positive Airway Pressure (CPAP). Similarly, the hazard of death among neonates with necrotizing enter colitis (NEC) was 2.78 times higher than neonates who did not have (NEC). This result is supported by a study conducted in Brazil [26], China [30], South Africa [31], and Bahir Dar, Ethiopia [12]. The reason for this could be the majority of the study participants were preterm, and all of them were LBW,these groups of neonates have immature gastrointestinal tract and immature defense mechanisms, so they are more susceptible to infection, and as the intestinal tracts colonize by microbes it could easily perforate and develops peritonitis [42]. In addition to the above reasons, neonates with NEC have intestinal necrosis and dehydration, and this may increase the chance of death. In the present study, neonates with birth weights, less than 1000 g and 1000–1499 g had three times and two times, higher risk of death as compared with neonates with a birth weight of 1500 to 2499.9 g respectively. This finding was consistent with studies conducted in Brazil [19], India [21], Bahir Dar, Ethiopia [12], and southern Ethiopia [16]. The possible justification for this could be, as birth weight decreases the risk of exposure to infection, hypothermia, and hypoglycemia could increase, and this could lead to the death of neonates. The other possible reason might be, due to immaturity of the lung which causes RDS, and it could lead to death. Particularly the immune system of LBW neonates may not be fully developed and may not fight of infections, therefore, it increases the hazard of death.

In this study, the other predictor of mortality was prematurity, which increases the hazard of death nearly two times as compared with those LBW neonates with a gestational age of 37 week and above. This finding was consistent with studies conducted in Brazil [27, 51], Iraq [29], Burkina Faso [28], Zimbabwe [25], and Southern Ethiopia [16]. The possible reason for this could be premature neonates have difficulty in adaptation of the extra uterine environment, due to immaturity of the organ system, and they face many fatal neonatal problems. This problem could decrease the probability of survival [8, 42]. Additionally, this may be due to improper care and inadequate availability of medical services and equipment like surfactant therapy, and mechanical ventilators unlike in middle-income and high-income countries.

### Limitation

This study was conducted using a secondary data source, important variables, such as maternal and paternal educational level, occupational status, nutritional status of the mother, and monthly income were missed. In addition to this, the present study was conducted in hospitals, where neonates who were in the community were not included, which may underestimate the mortality. On the other hand, only hospitalized (high-risk) neonates were included, and it may overestimate the mortality rate.

## Conclusion

In conclusion, the incidence of early neonatal mortality in LBW neonates was incredibly higher than e the overall early neonatal mortality in Northwest Ethiopia (Amhara region), and it continues to be a major public health problem. The highest number of death were recorded on the first day of admission. Preeclampsia, PNA, RDS, NEC, Birth weight, and prematurity were independent predictors of mortality among LBW neonates in their first week of life in Northwest Ethiopia.

Therefore, the stakeholders better to give strong emphasis to ELBW & VLBW neonates. The health care provider and other stakeholders shall give more emphasis and critical care on the first day of admission. Special care and support shall be given to LBW neonates who have fatal comorbidities like PNA, NEC, RDS, and neonates born from preeclampsia mothers. Further more, health care providers shall give appropriate ANC and PNC care and also they shall educate the mother and should give advice to the caregivers to perform KMC and breastfeed exclusively. A longitudinal prospective study in each low birth weight category will be better to address important variables that are missed in a retrospective study and to address the true effect of predictor variables.

### Electronic supplementary material

Below is the link to the electronic supplementary material.

Supplementary Material 1

Supplementary Material 2

## Data Availability

The dataset used and/or analyzed during the current study are available from the corresponding author on reasonable request.

## Abbreviations

AHR: Adjusted Hazard Ratio
ANC: Antenatal Care
APGAR: Appearance, Pulse, Grimace, Activity, Respiration
APH: Antepartum Hemorrhage
CSH: Comprehensive Specialized Hospital
ELBW: Extremely Low-Birth-Weight
ENM: Early Neonatal Mortality
FMoH: Federal Ministry of Health
HIV/AIDS: Human Immunodeficiency Virus/Acquired Immune Deficiency Syndrome
IUGR: Intra-Uterine-Growth-Restriction
LBW: Low-Birth-Weight
NEC: Necrotizing Enterocolitis
NICU: Neonatal Intensive Care Unit
PNA: Perinatal Asphyxia
RDS: Respiratory Distress Syndrome
UoGCSH: University of Gondar Comprehensive Specialized Hospital
VLBW: Very Low-Birth-Weight
