# Supplementary material for: Incidence and predictors of mortality among low birth weight neonates in the first week of life admitted to the neonatal intensive care unit in Northwestern Ethiopia comprehensive specialized hospitals, 2022. Multi-center institution-based retrospective follow-up study
Source: BMC Pediatr. 2023 Sep 27;23:489. doi: 10.1186/s12887-023-04319-0 (PMC10523684; doi:10.1186/s12887-023-04319-0)
Supplement: Supplementary file 1 — Supplementary Material 1 [file 12887_2023_4319_MOESM1_ESM.docx]

**Data abstraction checklist**

Check list to assess Incidence and predictors of mortality among low-birth weight neonates in the first week of life admitted to West Amhara region Comprehensive Specialized Hospital, Northwest Ethiopia, 2022.

**Instructions:** Circle the possible responses or write them in the space provided corresponding to each question.

Hospital name ----------------------------------------------

Name of data collector--------------------------- signature--------------------

Name of supervisor---------------------------------signature---------------------

Code of the questionnaires.………………………..

**Part I: Socio-demographic characteristics of parent-neonate pairs**

| Code No | Variables | Response | Remark |
| --- | --- | --- | --- |
| 101 | Age of the mother | ––––––––– in yr. |  |
| 102 | Sex of the neonate | 1. Male 2. Female |  |
| 103 | Age of the neonate on admission | ––––––––––––––in day. |  |
| 104 | Residence |  |  |
| 105 | Place of delivery | 1. Health institution  2. Out of Health institution |  |

Part II; **Obstetric and gynecological related predictors**

| Code No | Variables | Response | Remark |
| --- | --- | --- | --- |
| **201** | Does the mother have ANC follow up | 1. Yes 2. No |  |
| **202** | Mode of delivery | 1.Spontaneous Vaginal Delivery (SVD)  2.Instrumental 3.C/S |  |
| **203** | Type of pregnancy | 1. Single 2. Multiple |  |
| **204** | Gravidity in number |  |  |
| **205** | Parity in number? |  |  |
| **206** | Does the mother take corticosteroid treatment in the last pregnancy? | 1. Yes 2. No |  |
| **207** | Does the mother have Preeclampsia | 1. Yes  2. No |  |
| **208** | Does the mother have a history of PROM in the last pregnancy? | 1. Yes 2. No |  |
| **209** | Did the mother have bleeding during pregnancy (APH) in the last pregnancy? | 1. Yes  2. No |  |
| **Part III: Maternal medical factors** | | | |
| **301** | Did the mother have CHTN | 1. Yes 2. No |  |
| **302** | Maternal DM | 1. Yes 2. No |  |
| **303** | Maternal HIV infection | 1. Yes 2. No |  |
| **304** | Maternal Anemia | 1. Yes 2. No |  |
| **305** | Maternal Tuberculosis | 1. Yes 2. No |  |
| **306** | Other maternal comorbid illness (UTI, STD) |  |  |

Part IV: Clinical related variables among low birth weight neonates.

| Code no | variables | Response | Remark |
| --- | --- | --- | --- |
| 401 | First minute APGAR score |  |  |
| 402 | 5 ^th^ minute APGAR score |  |  |
| 403 | Birth weight (in gram). | –––––––. |  |
| 404 | Gestational age of the neonate? | –––––in week/day. |  |
| 405 | Breast feeding | 1. Yes   2. No |  |
| 406 | Any comorbid neonatal illnesses? (Multiple responses) | 1. PNA. 2. Sepsis 3. RDS 4. Jaundice 5. NEC 6. Congenital anomalies 7. Hypo/hyperthermia 8. IUGR 9. Other…… |  |
| 4011 | Random blood sugar at admission in mg/dl |  |  |
| Part IV: Outcome related variables (Follow-up measures). | | | |
| 501 | Date of admission to NICU in DD/MM/Yr. in G.C | –––––/–––––/––––––– G.C |  |
| 502 | Follow up outcome of the neonate | 1. Died  2. Discharge  3.Transfered in / referred  4. Left against medical advice  5. Lost to follow up  6. End of follow uptime | 1.If died go to 503  2. If not died go to 505 |
| 503 | Date of death DD/MM/YY in G.C |  |  |
|  | If died, age at death in day? |  |  |
| 505 | Date of discharge DD/MM/YY in G.C? | ––––––––in day. |  |
| 506 | Date of Left against medical advice DD/MM/YY in G.C. | –––/––––/–––– |  |
| 507 | Referred out DD/MM/YY in G.C | ­­–––/––––/–––– |  |
| 508 | Date of lost to follow-up | –––/––––/–––– |  |
| 509 | Survived beyond the follow-up DD/MM/YY in G.C | –––/––––/––– |  |
| 510 | At what age censored | ––––––––in a day. |  |
| 5011 | Total no of days the neonate has been followed? | ––––––––––in days |  |
