# Supplementary figures and images for: Incidence and predictors of mortality among low birth weight neonates in the first week of life admitted to the neonatal intensive care unit in Northwestern Ethiopia comprehensive specialized hospitals, 2022. Multi-center institution-based retrospective follow-up study
Source: BMC Pediatr. 2023 Sep 27;23:489. doi: 10.1186/s12887-023-04319-0 (PMC10523684; doi:10.1186/s12887-023-04319-0)

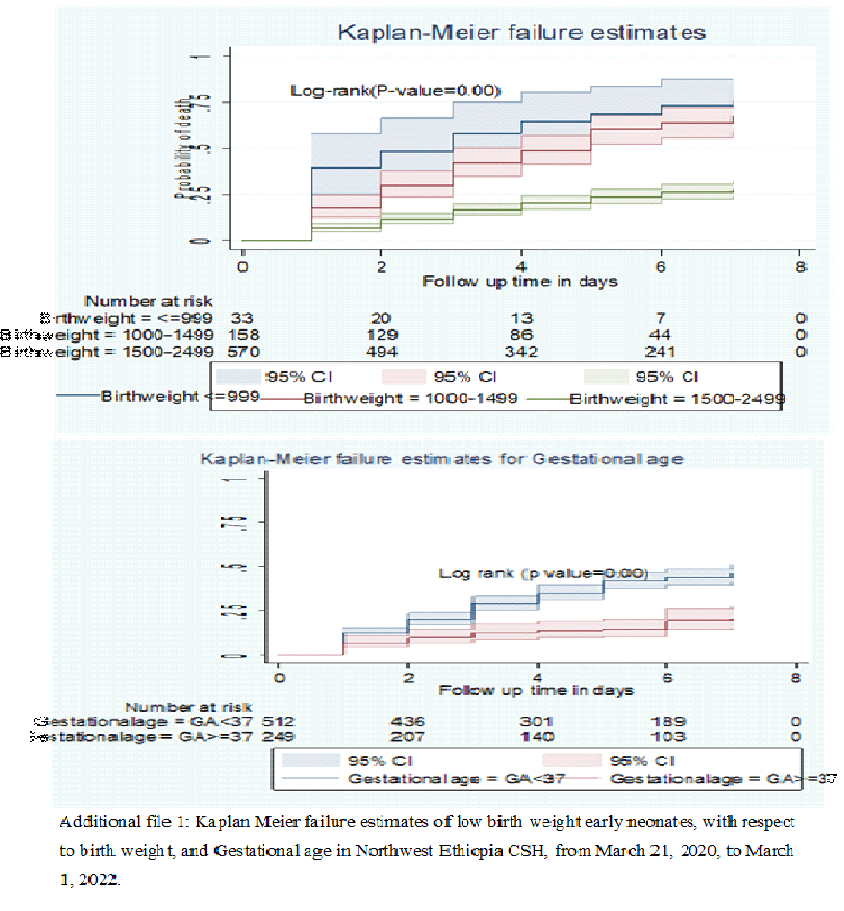

Supplement: Supplementary file 2 — Supplementary Material 2 [file 12887_2023_4319_MOESM2_ESM.tiff]
